# Supplementary material for: Treatment of necrotizing enterocolitis by conditioned medium derived from human amniotic fluid stem cells
Source: PLoS One. 2021 Dec 2;16(12):e0260522. doi: 10.1371/journal.pone.0260522 (PMC8638898; doi:10.1371/journal.pone.0260522)
Supplement: Attachment — Submitted filename: Review.docx [file pone.0260522.s002.docx]

Joshua S. O’Connell et al. present a research on therapeutic effect of conditioned medium (CM) derived from human AFSC on NEC without risk of tumorgenesis as well as immunogenicity. It is quite interesting but remain some problems. Detailed comments are as follows:

1. In line 61, what kinds of MSC does the author refer to? As postnatal MSC contain various types of MSC such as bone marrow-derived or adipose-derived mesenchymal stem cells.

2. Flow chart of the experiment is suggested.

3. Why does the author choose 100μL as the dose of CM to treat NEC? The author should explain.

4. Is there any difference of therapeutic efficacy between CM and MSC transplantation?

5. The author should add more experiments to examine the effect of CM on NEC such as apotosis or others.

6. As the effect of CM was so prominent, the author need to explain what composition in CM plays an key role in the CM-mediated therapeutic effect.

.
