# Supplementary material for: Treatment of necrotizing enterocolitis by conditioned medium derived from human amniotic fluid stem cells
Source: PLoS One. 2021 Dec 2;16(12):e0260522. doi: 10.1371/journal.pone.0260522 (PMC8638898; doi:10.1371/journal.pone.0260522)
Supplement: Attachment — Submitted filename: Response to Reviewers PONE-D-21-06129.docx [file pone.0260522.s003.docx]

**Response to Reviewers’ Comments**

**Treatment of necrotizing enterocolitis by conditioned medium derived from human amniotic fluid stem cells**

We would like to thank the reviewers for their careful review of our manuscript, and their thoughtful and constructive comments. Our responses to the reviewers’ comments are in blue in this letter. The corresponding modifications in the manuscript are highlighted in yellow.

Reviewer #1:

Joshua S. O’Connell et al. present a research on therapeutic effect of conditioned medium (CM) derived from human AFSC on NEC without risk of tumorgenesis as well as immunogenicity. The manuscript must describe a technically sound piece of scientific research with data that partly supports the conclusions. Experiments must have been conducted rigorously, with appropriate controls, replication, and sample sizes. The conclusions must be drawn appropriately based on the data presented. It is quite interesting but remains some problems that needs to be addressed.

Detailed comments are as follows:
1. In line 61, what kinds of MSC does the author refer to? As postnatal MSC contain various types of MSC such as bone marrow-derived or adipose-derived mesenchymal stem cells.

Response:

We agree with the reviewer that this needed to be clarified. The postnatal MSCs we refer to are bone marrow derived. This change has been made in the introduction of the manuscript (Line 61).

2. Flow chart of the experiment is suggested.

Response:

Thank you for this suggestion. A flow chart has been added to the discussion of the manuscript.

3. Why does the author choose 100μL as the dose of CM to treat NEC? The author should explain.

Response:

The chosen dose of 100μL is based on previous publications of conditioned media from human amniotic fluid-derived stem cells (hAFSC-CM) and their therapeutic effects in experimental mice models^1,2^. This information has been added to the materials and methods of the manuscript (Lines 137-139).

4. Is there any difference of therapeutic efficacy between CM and MSC transplantation?

Response:

There is no difference of therapeutic efficacy between CM and MSC transplantation. Similar to our results, it has been reported in experimental NEC that IP injection of bone marrow-derived mesenchymal stem cells (BM-MSC) and amniotic fluid-derived mesenchymal stem cells (AF-MSC) significantly reduced NEC incidence and morphological damage^3,4^. As demonstrated in this experimental NEC study, AFSC-CM administration reduced NEC incidence and morphological damage, inflammation (myeloperoxidase), and cell apoptosis (cleaved caspase 3), as well as increased intestinal proliferation and stem cell activation. These results are similar to what was found with IP injection of AFSCs in experimental NEC^5^.

5. The author should add more experiments to examine the effect of CM on NEC such as apotosis or others.

Response:

We have performed additional experiments to study the beneficial effects of CM on the intestinal inflammation, apoptosis, and angiogenesis. This information has been added to the manuscript (Lines 221-235).

6. As the effect of CM was so prominent, the author need to explain what composition in CM plays an key role in the CM-mediated therapeutic effect.

Response:

We have performed additional experiments to study the proteomics of the AFSC-CM. This information has been added to the manuscript (Lines 269-275).

Reviewer #2:

This manuscript by O’Connell JS et al. describes the protective effects of conditioned medium derived from human amniotic fluid stem cells (AFSC) in experimental mouse model of necrotizing enterocolitis (NEC). NEC was induced starting at post-natal day P5 via gavage feeding of hyperosmolar formular 4x a day, immediately following 10 minute hypoxia prior to all feeds and LPS administration on P6 and P7. For treatment, CM were administered on P6 and P7 at a dose of 100uL/each via IP injection; PBS administration were used as control. The experiment was terminated 2 days after the last administration of CM at post-natal day P9, and terminal ileum were harvested and processed for histologic evaluation and molecular characterization for IL-6, TNFalpha, Lgr5 gene expression. The authors concluded that the CM treatment group demonstrated less tissue damage, lower pro-inflammatory IL-6 and TNF-alpha expression and improved counts for stem cell activity marker Lgr5. The manuscript is well written and the research study address an important problem in neonatal gastroenterology. Knowledge gained from this study could potentially advance the field.

Major concerns:

1. The authors have previously published (ref. #14) that human AFSC derived extracellular vesicles provide benefits identical to the results reported herein Are the AFSC used in this study different from that used in reference #14, and if so, how? The authors refer to ref. #14 in relation to methods used and in the context of EVs in general as a potential mechanism of action in the CM, but the discussion falls short of clearly stating that those EVs were also derived from AFSC. It should be therefore clearly stated in the discussion how these two studies are different in terms of the stem cells used (if they are), otherwise it seems as if the hypothesis proposed herein has been previously tested. If the stem cells in both studies are of the same kind (not necessarily from the same source), then there is no novelty and/or significance to the present study, as in some respects it replicates previous work.

Response:

We fully agree with the reviewer’s comments. As we previously demonstrated the AFSC derived EVs could be beneficial in reducing the NEC induced intestinal damage^6^, which is similar to the AFSC CM effects we found in this study. However, the AFSC-CM contains components not limited exclusively to EVs, but also proteins, indicating a broader effect than exclusive AFSC EVs administration. That is the rational for our current study to lay down the foundation to mining the beneficial component from AFSC CM. This information has been added to the manuscript (lines 345-349).

2. The data for gene expression for IL6, TNFa and Lgr5 are impressive, however, confirmation of this effect at protein level will be more convincing. VEGF is known to be decreased during NEC; have you assessed if treatment with CM helps restore VEGF levels? How about neutrophils and macrophages?

Response:

We have performed additional experiments to study the beneficial effects of CM on intestinal inflammation, neutrophil infiltration, apoptosis, and angiogenesis. This information has been added to the manuscript (Lines 221-235).

3. Also, adding an immunostaining for Lgr5 or rather a co-staining of Lgr5 and Ki67 together will be more valuable and informative to demonstrate intestinal repair and regeneration.

Response:

Immunostaining for Lgr5 is difficult due to no validated specific antibody. Instead, we performed co-staining for intestinal stem cell marker Olfm4^7^, and epithelial proliferation marker Ki67 to demonstrate the intestinal regeneration ability. The new experimental data has been added this manuscript (Lines 248-257)

4. How was it determined that two doses of CM administration were needed to generate the beneficial effect? Is there evidence that single dose is not effective?

Response:

We agree with the reviewer that this needs to be clarified. The two-dose administration was based on previous publications that showed therapeutic effects of IP injected AFSC^5^ and AFSC extracellular vesicles^8^ in experimental NEC. Two doses via similar administration of IP injection allows for comparison with respect to potential treatment.

This information has been added to the materials and methods of the manuscript (Lines 137-139).

5. Does the CM treatment provide long term benefit, or the observed changes are transient? In this regard, a survival study will be very useful for demonstrating any lasting effects.

Response:

We have performed additional experiments to study the mice pup’s survival. This new data has been added to the manuscript (Lines 211-215).

Minor concerns:

1. DAPI staining (described in methods) is missing in Figure 4.

Response:

This information has been added to Figure 4 of the manuscript.

**References for the response for the reviewers:**

1.	Jun EK, Zhang Q, Yoon BS, et al. Hypoxic conditioned medium from human amniotic fluid-derived mesenchymal stem cells accelerates skin wound healing through TGF-β/SMAD2 and PI3K/Akt pathways. Int J Mol Sci. 2014;15(1):605-628. Published 2014 Jan 6.

2.	Mellows B, Mitchell R, Antonioli M, et al. Protein and Molecular Characterization of a Clinically Compliant Amniotic Fluid Stem Cell-Derived Extracellular Vesicle Fraction Capable of Accelerating Muscle Regeneration Through Enhancement of Angiogenesis. Stem Cells Dev. 2017;26(18):1316-1333.

3.	McCulloh, C. J., Olson, J. K., Zhou, Y., Wang, Y., & Besner, G. E. Stem cells and necrotizing enterocolitis: A direct comparison of the efficacy of multiple types of stem cells. Journal of Pediatric Surgery. 2017;52(6):999–1005.

4.	Tayman, C., Uckan, D., Kilic, E., Ulus, A. T., Tonbul, A., Murat Hirfanoglu, I., Helvacioglu, F., Haltas, H., Koseoglu, B., & Tatli, M. M. Mesenchymal Stem Cell Therapy in Necrotizing Enterocolitis: A Rat Study. Pediatric Research. 2011;70(5):489–494.

5.	Zani A, Cananzi M, Fascetti-Leon F, et al. Amniotic fluid stem cells improve survival and enhance repair of damaged intestine in necrotising enterocolitis via a COX-2 dependent mechanism. Gut. 2014;63(2):300-309.

6.	O’Connell JS, Lee C, Farhat N, Antounians L, Zani A, Li B, Pierro A. Administration of extracellular vesicles derived from human amniotic fluid stem cells: a new treatment for necrotizing enterocolitis. Pediatr Surg Int. 2021.

7.	van der Flier LG, Haegebarth A, Stange DE, van de Wetering M, Clevers H. OLFM4 is a robust marker for stem cells in human intestine and marks a subset of colorectal cancer cells. Gastroenterology. 2009;137(1):15-17.

8.	Li B, Lee C, O'Connell JS, Antounians L, Ganji N, Alganabi M, Cadete M, Nascimben F, Koike Y, Hock A, Botts SR, Wu RY, Miyake H, Minich A, Maalouf MF, Zani-Ruttenstock E, Chen Y, Johnson-Henry KC, De Coppi P, Eaton S, Maattanen P, Delgado Olguin P, Zani A, Sherman PM, Pierro A. Activation of Wnt signaling by amniotic fluid stem cell-derived extracellular vesicles attenuates intestinal injury in experimental necrotizing enterocolitis. Cell Death Dis. 2020 Sep 14;11(9):750.
