# Supplementary material for: Treatment of necrotizing enterocolitis by conditioned medium derived from human amniotic fluid stem cells
Source: PLoS One. 2021 Dec 2;16(12):e0260522. doi: 10.1371/journal.pone.0260522 (PMC8638898; doi:10.1371/journal.pone.0260522)
Supplement: Attachment — Submitted filename: Response to Reviewers PONE-D-21-06129 R2.docx [file pone.0260522.s004.docx]

**Response to Reviewers’ Comments**

**Treatment of necrotizing enterocolitis by conditioned medium derived from human amniotic fluid stem cells**

We would like to thank the reviewers for their careful review of our manuscript, and their thoughtful and constructive comments. Our responses to the reviewers’ comments are in blue in this letter. The corresponding modifications in the manuscript are highlighted in yellow.

Reviewer #2: This revised manuscript shows an improvement over the original submission, and the new data provided greatly enhances the interpretation of the results. However, the manuscript will further benefit by addressing the following:

1. Figure 2F-G are not referenced in the text.

Response:

Figure 2 H and I are the quantifications of Figure 2 F and G. We have updated our text (Line 228 and 232).

2. Grammatical error in the following lines 230-232

Response:

Thank you for highlighting this error. We have updated our text, lines 230-232.

3. Figure 2J is not referenced. The VEGF expression data is missing in the results. What is the conclusion from the VEGF data is not mentioned throughout the manuscript.

Response:

We have included the result of the VEGF expression in the “Result” and “Discussion” sections. See lines 233-235 and lines 289, 330-332

4. The DAPi staining in Figure 3C for the NEC +PBS group does not match with the rest of the stainings.

Response:

Figure 3C and D has been updated.

5. The sample size for the NEC + PBS group needs clarification. It is n=11 according to the dot plot in Figure 3E, but the legend states n=10. This needs to be clarified also in the methods, line 141-142.

Response:

Thank you for pointing out this mistake. We have updated the NEC+PBS group in Figure 3E.

6. Figure 5 illustration can be improved by providing the following:

a. description of the cells types shown

b. change of gene or pathway expression in the NEC +CM group similar to the NEC (or NEC + PBS)

Response:

Figure 5 has been updated to include the description of cell types and the pathways expressed in the NEC+PBS and NEC+CM groups.
